# Generation of H9 T-cells stably expressing a membrane-bound form of the cytoplasmic tail of the Env-glycoprotein: lack of transcomplementation of defective HIV-1 virions encoding C-terminally truncated Env

**DOI:** 10.1186/1742-4690-3-27

**Published:** 2006-05-16

**Authors:** Denise Holtkotte, Tanya Pfeiffer, Valerie Bosch

**Affiliations:** 1 Forschungsschwerpunkt Infektion und Krebs, F020, Deutsches Krebsforschungszentrum, Im Neuenheimer Feld 242, 69120 Heidelberg, Germany

## Abstract

H9-T-cells do not support the replication of mutant HIV-1 encoding Env protein lacking its long cytoplasmic C-terminal domain (Env-CT). Here we describe the generation of a H9-T-cell population constitutively expressing the HIV-1 Env-CT protein domain anchored in the cellular membrane by it homologous membrane-spanning domain (TMD). We confirmed that the Env-TMD-CT protein was associated with cellular membranes, that its expression did not have any obvious cytotoxic effects on the cells and that it did not affect wild-type HIV-1 replication. However, as measured in both a single-round assay as well as in spreading infections, replication competence of mutant pNL-Tr712, lacking the Env-CT, was not restored in this H9 T-cell population. This means that the Env-CT *per se *cannot transcomplement the replication block of HIV-1 virions encoding C-terminally truncated Env proteins and suggests that the Env-CT likely exerts its function only in the context of the complete Env protein.

## Findings

In contrast to most other enveloped viruses, the surface Env glycoproteins of lentiviruses, including HIV-1, contain very long C-terminal cytoplasmic tails (CTs). In the case of HIV-1, the Env-CT has a conserved length of about 150 amino acids (aa) and mutant viruses, encoding truncated Env proteins, are unable to replicate in most T cell-lines [1]. However, despite its undoubted importance for the HIV-1 life cycle, the biological mechanism by which the long Env-CT facilitates virus replication is still not fully understood. Numerous studies employing HIV Env-CT mutants have addressed the potential roles of various motifs and features within the Env-CT. Of relevance for this study is the fact the Env-CT may impact cellular phenomena. Thus, for example, the HIV-Env-CT has been reported to bind to calmodulin and to inhibit calmodulin-regulated proteins [2,3]. Furthermore, yeast 2 hybrid screenings have identified further potential cellular interaction partners of the HIV-Env-CT. These are α-catenin, which is involved in cellular adhesion [4,5], and p115-RhoGEF, which regulates actin stress fiber formation and activates the serum response factor (SRF) [6]. It is possible that these interactions with cellular processes, or others presently unknown, are important for viral replication. For example, it is conceivable that the Env-CT itself accesses signal transduction pathways to alter cellular gene expression and facilitate virus replication or, alternatively, membrane-bound Env-CT itself may recruit essential cellular proteins to cellular membranes sites and thus facilitate virus assembly and release.

In this study, we have generated and characterised H9 T-cells which stably express a membrane-bound version of the Env-CT and examined if the presence of this region *alone *might be sufficient for the transcomplementation of originally non-infectious HIV-1 virions encoding for truncated Env glycoproteins.

**Figure 1:**
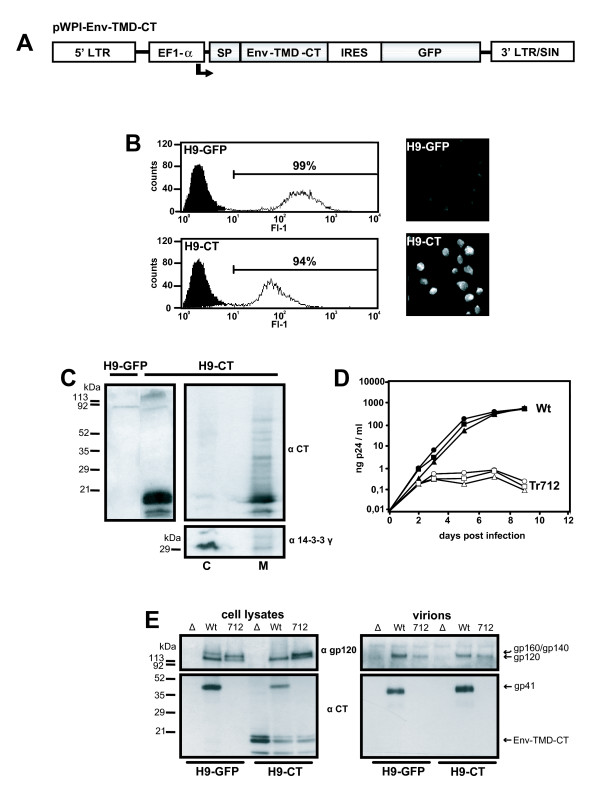
Generation of, and virus replication in, an H9 T-cell population stably expressing HIV-Env-TMD-CT. A. schematic representation of the SIN lentiviral vector pWPI-Env-TMD-CT employed. An internal human EF1-α promoter drives expression of a transcriptional unit consisting of the Env-TMD-CT gene, an IRES element and the gene for GFP. The composition of the Env-TMD-CT gene is described in the text. SP; signal peptide sequence of tissue plasminogen activator, Env-TMD; membrane anchor of HIV-Env, CT; cytoplasmic domain of HIV-Env. B. FACS for GFP expression (left panels) and indirect immunofluorescence analyses for Env-TMD-CT expression (right panels) of H9 T-cell populations stably transduced with pWPI (H9-GFP) and pWPI-Env-TMD-CT vector particles (H9-CT). Immunofluorescence of paraformaldehyde-fixed permeabilised H9 cells was performed with rabbit anti-gp160 serum shown to contain antibodies against the Env CT, followed by biotinylated goat anti-rabbit IgG and streptavidin phycoerythrin. Identical exposure times were used to generate the images from the H9-GFP and the H9-CT cells. C. Western blot analysis of H9 cells stably transduced with pWPI (H9-GFP) and pWPI-Env-TMD-CT (H9-CT) (left panel) and of cytosolic (C) and membrane (M) fractions of H9-CT cells (right panel) with gp41 Mab Chessie 8 [16] as indicated. After stripping, the right blot was reprobed with rabbit antibodies specific for the cytoplasmic protein 14-3-3 γ (C-16) (Santa Cruz Biotechnology). D. Replication kinetics of Wt-pNL-4-3 (Wt) (filled-in symbols) and pNL-Tr712 virions (Tr712) (empty symbols) in H9 cells (circles), H9-GFP cells (triangles) and H9-CT cells (squares). Infections were initiated with 100 ng virus per 10^6 ^cells, produced by transfection of the respective plasmids in 293T cells. 5 h p.i., the cells were thoroughly washed and the course of infections followed by measurement of newly released HIV-CA in the supernatant by ELISA. E. Western blot analyses of equalised amounts (by CA-ELISA of culture supernatants) of lysates of H9-GFP and H9-CT cells infected with pNL-Δ Env (Δ), pNL-Wt (Wt) and pNL-Tr712 (712) (left) and of equalised amounts (by CA-ELISA of ultracentrifuged particles) of the respective virions released into the media (right). The top portions of the filters have been probed with anti-gp120 serum and the bottom portion with anti-CT antibodies (Chessie 8).

pWPI-Env-TMD-CT, depicted in Fig 1A, encodes a membrane-bound form of the Env-CT and is based on the bicistronic lentiviral vector pWPI (obtained from D. Trono, University of Geneva, Switzerland). pWPI-Env-TMD-CT and pWPI additionally express green fluorescent protein (GFP) downstream of an internal ribosomal entry site (IRES). The Env-TMD-CT gene consists of the signal peptide (SP) sequence from tissue plasminogen activator (tPA) (tPA amino acids (aa) 1–35) fused via a 4aa spacer to the membrane-spanning (TMD) and CT domains of the HIV BH10-Env protein (aa 684–851). The 4aa spacer consists of 2 HIV-Env aa (Thr, Glu) C-terminal to the HIV-SP cleavage site and 2 HIV-Env aa N-terminal to the TMD (Ile, Lys).

Lentiviral vector particles were generated by cotransfection of 293T cells with pWPI-Env-TMD-CT or, as a control, pWPI plus the packaging construct pCMVΔR8.91 [7] and the VSV-G expression plasmid pMD.G [8]. Vector particles, concentrated from the culture supernatant by ultracentrifugation, were employed to transduce H9 cells. Transduced cell populations were then sorted for maximum GFP expression and expanded. The resulting transduced populations were designated H9-CT cells (expressing Env-TMD-CT and GFP) and H9-GFP cells (expressing only GFP). As shown in Fig. 1B, both populations were over 90% positive for GFP expression although clearly the fluorescence intensity of the H9-CT population was lower than that of the H9-GFP population. This may be a result of GFP expression being decreased when preceded by the Env-TMD-CT gene. Expression of the Env-TMD-CT protein in the sorted H9-CT population was first analysed by indirect immunofluorescence of paraformdehyde-fixed, permeabilised cells employing rabbit anti-gp160 serum which we have previously demonstrated to contain antibodies against the Env-CT. As shown in Fig. 1B, right panels, in comparison to H9-GFP cells, virtually all of the cells in the H9-CT culture were positive for Env-TMD-CT expression. Western blot analysis confirmed expression of a specific Env-TMD-CT protein band migrating at about the position of its calculated molecular weight (18.8 kDa after removal of the SP) (Fig. 1C, left panel). In addition, some minor species migrating slightly slower or faster than the major Env-TMD-CT protein, which may represent species still containing the SP or degradation products, respectively, were detectable. In order to confirm localisation to cellular membranes, cytosolic (C) and membrane (M) fractions from H9-CT cells were prepared employing published procedures [9]. Western blot analysis of equivalent amounts of these fractions demonstrated that the Env-TMD-CT protein was localised predominantly in the membrane fraction and only a minor amount remained in the cytosolic fraction (Fig. 1C, right panels). Reprobing the blot with antibodies to the 30 kDa cytosolic protein 14-3-3 γ (C-16) [10] confirmed the authenticity of the membrane/cytosol separation. In summary, these results point to functional membrane insertion of the Env-TMD-CT protein.

There were no obvious cytotoxic effects on the H9-CT cells as a result of expression of the Env-TMD-CT protein. Thus cell growth was not reduced in comparison to H9-GFP cells and cell morphology was unaffected (data not shown). We then went on to examine the replication of wild-type HIV (pNL4-3, referred to as pNL-Wt) and mutant pNL-Tr712, encoding truncated Env protein in which only 7aa of the 151 aa long Env-CT remain [11], in H9, H9-GFP and H9-CT cells. The viruses were generated by transfection of the respective proviral plasmids in 293T cells and amounts equivalent to 100 ng CA/10^6 ^cells, as determined by enzyme-linked immunosorbant assay (ELISA) (Innogenetics, Ghent, Belgium) were used to initiate infection of the H9 cell populations. After removing input virus and thorough washing, the course of the infections was monitored by determining, via CA-ELISA, the amounts of released virions in the respective culture supernatants over time. As shown in Fig. 1D, pNL-Wt virus replicated efficiently in all the H9 cell populations but with a slight delay in both H9-CT and H9-GFP cells. The basis for this slight delay in replication kinetics is not known. As had been shown previously [1], pNL-Tr712 cannot give rise to a spreading infection in H9 cells nor, as to be expected, was this the case in H9-GFP cells. In the H9-CT cell population, spreading infection of pNL-Tr712 virus also does not occur despite the presence of the Env-CT region anchored at the cellular membrane by its homologous TMD.

In order to generate virions for further analyses, the respective H9 cell populations were infected with VSV-G pseudotyped pNL-Wt, pNL-Tr712 virions and pNL-Δ Env virions using procedures previously described [12] and, after removal of input virus, the respective newly generated virions were collected. The infectivities of the virions in the supernatants of the infected H9 cells were analysed in a single-round assay in Tzm-bl reporter cells [13-15]. pNL-Tr712 virions exhibited reduced but still significant infectivity in comparison to pNL-Wt virions but the extent of the reduction was independent of whether the virions were produced in H9-GFP cells or in H9-CT cells expressing membrane-bound Env-TMD-CT (data not shown). This shows that the expression of the Env-TMD-CT protein in producer H9 T-cells does not result in an increase of the infectivity of released pNL-Tr712 virions. In order to examine if the Env-TMD-CT protein, expressed in the H9-CT cells, was incorporated into released virions, the respective virions were concentrated by ultracentrifugation from the media of infected H9-CT cells or H9-GFP cells and lysates of infected cells and virions examined in Western blot (Fig. 1E). Virally-expressed gp160, gp120 and gp41 were detectable in lysates of pNL-Wt infected cells and truncated gp160 (gp140) and gp120 in lysates of pNL-Tr712 infected cells. The truncated gp41 species (gp28) expressed by pNL-Tr712 was not detected since the antibodies employed (Chessie 8 [16]) bind to an epitope in the Env-CT missing in this protein. In the lysates of all the infected H9-CT cultures, constitutively expressed Env-TMD-CT protein was detectable. Its expression level was similar to that of the gp41 protein expressed after infection with pNL-Wt. In Fig. 1E, right panel, analysis of equalised amounts (by CA-ELISA) of virions concentrated from the supernatants of the respective infected cultures is shown. Gp120 and gp41 proteins were detectable in pNL-Wt virions and gp120 protein was detectable in pNL-Tr712 virions (again the truncated gp28 band cannot be detected). This observation of gp120 incorporation into pNL-Tr712 virions stands in contrast to two studies in the literature [1,17] which report that Env incorporation into pNL-Tr712 virions is defective when these are produced in non-permissive cells. However, we consistently observe gp120 incorporation into pNL-Tr712 and have recently reported that this is also the case with another mutant HIV encoding Env with a different C-terminal truncation [12]. The reason for this discrepancy is presently unknown. Of interest in the context of this report is the fact that, although the respective virions have incorporated gp120/gp41, the constitutively expressed Env-TMD-CT protein was not detectable in any of the released virions. The phenomena which determine if particular cellular and viral proteins are incorporated into virions or not are not understood in depth. Thus also in this case, we can only speculate that perhaps the Env-TMD-CT protein may not be localised at the cellular sites of virus assembly or may not appropriately interact with cellular proteins influencing localisation/incorporation.

In summary, in this report we describe a cell population in which the majority, and likely all of the cells express a native i.e. untagged version of the HIV-Env-CT domain anchored in their cellular membranes by its homologous membrane anchor. We envisage that the expressed Env-TMD-CT protein likely adopts its native conformation although we cannot formally rule out the possibility that this may require the presence of the Env ectodomain. The presence of the membrane-bound Env-TMD-CT protein in the H9 cells was not sufficient to transcomplement the replication block of virions encoding C-terminally truncated Env proteins. Although other reasons may account for this lack of transcomplementation, the most likely explanation is that the Env-TMD-CT has to be part of the full-length Env protein in order to fulfill its essential function(s). Nevertheless, H9 CT cells (and control H9-GFP cells) may still be useful tools to study possible effects of the Env-TMD-CT protein on cellular processes such as signal transduction phenomena/cellular gene expression.

## Competing interests

The author(s) declare that they have no competing interests.

## Authors' contributions

DH carried out the replication kinetics, participated in the cloning of pWPI-TMD-Env-CT and the generation of the stable H9 cell lines and was involved in drafting the manuscript. TP carried out FACS, Western blot and membrane fractionation analyses. VB participated in the design of the study and in drafting the manuscript. All authors read and approved the final manuscript.
